# Supplementary material for: SARS-CoV-2 Infection Is an Independent Risk Factor for Decompensation in Cirrhosis Patients
Source: Diseases. 2024 Feb 25;12(3):46. doi: 10.3390/diseases12030046 (PMC10968826; doi:10.3390/diseases12030046)
Supplement: Supplementary file 1 [file diseases-12-00046-s001.zip › diseases-2866557-supplementary.pdf]

Supplementary Materials of Methods:

Table S1. ICD-10 Codes Used in Methodology.

• ***Patient Diagnoses***		
Compensated Cirrhosis	Alcoholic cirrhosis of liver without ascites	K70.30
	Hepatic fibrosis	K74
	Alcoholic cirrhosis of liver	K70.3
	Primary biliary cirrhosis	K74.3
	Other cirrhosis of liver	K74.69
	Unspecified cirrhosis of liver	K74.60
	Fibrosis and cirrhosis of liver	K74
	Secondary biliary cirrhosis	K74.4
	Biliary cirrhosis, unspecified	K74.5
	Alcoholic fibrosis and sclerosis of liver	K70.2
• ***Outcomes***		
Decompensation (Any of the following)	Esophageal varices without bleeding	I85.00
	Hepatic failure, unspecified with coma	K72.91
	Hepatic encephalopathy	K76.82
	Esophageal varices with bleeding	I85.01
	Spontaneous bacterial peritonitis	K65.2
	Secondary esophageal varices	I85.1
	Esophageal varices	I85.0
	Esophageal varices	I85
	Ascites	R18
	Hepatorenal syndrome	K76.7
	Other ascites	R18.8
	Alcoholic cirrhosis of liver with ascites	K70.31
Ascites	Ascites	R18
	Other ascites	R18.8
	Alcoholic cirrhosis of liver with ascites	K70.31
HRS	K76.7	
HE	Encephalopathy, unspecified	G93.40
	Hepatic encephalopathy	K76.82
	Hepatic failure, unspecified with coma	K72.91
SBP	K65.2	
Jaundice	R17	
Variceal Bleed	Gastric varices	I86.4
	Gastrointestinal hemorrhage, unspecified	K92.2
• ***Propensity Score Matching Components***		
Coronary Artery Disease	I25.1	
Essential Hypertension	I10	
Diabetes	E08-E13	
Chronic Kidney Disease	N18	
Chronic Obstructive Lung Disease	J44	
